# Early Changes in House Dust Mite Component Specific Immunoglobulin Levels Predict the One‐Year Efficacy of Allergen Immunotherapy in Patients With Allergic Rhinitis

**DOI:** 10.1002/clt2.70099

**Published:** 2025-09-08

**Authors:** Shuying Li, Yue Ma, Jiaying Li, Xian Feng, Fang Xiong, Miaomiao Han, Huabin Li, Hongfei Lou

**Affiliations:** ^1^ Department of Otolaryngology Eye & ENT Hospital Fudan University Shanghai China

**Keywords:** allergen immunotherapy, allergic rhinitis, component‐resolved diagnostics, house dust mite, prediction model

## Abstract

**Background:**

The efficacy of subcutaneous immunotherapy (SCIT) in allergic rhinitis (AR) patients varies. Component‐resolved diagnostics (CRD) may serve as a useful tool to predict therapeutic responses.

**Methods:**

Forty‐three house dust mite (HDM)‐sensitized AR patients undergoing SCIT were enrolled. Clinical data and serum samples were collected at baseline (V1), 15 weeks (V2), and 1 year (V3). The levels of specific immunoglobulin E (sIgE) and sIgG4 to nine HDM components were measured, and a predictive model was established. An independent prospective cohort of 23 patients was used for validation.

**Results:**

The most prevalent sensitizing components were *Dermatophagoides pteronyssinus* (Der p) 1*, Dermatophagoides farinae* (Der f) 1, Der p 2, Der f 2, and Der p 23. The responders had a significantly higher Combined Symptom and Medication Score (CSMS) at baseline than did the nonresponders. At V2, the responders showed a greater increase in serum levels of Der f 1 sIgE, higher levels of Der p 23 sIgG4, and greater incremental changes in Der p 23 sIgG4 levels. A composite model based on V1 CSMS, ∆_15w_ Der f 1 sIgE, and ∆_15w_ Der p 23 sIgG4 achieved an area under the receiver operating characteristic curve (AUC) of 0.896 (cutoff: 0.455), with 83.3% sensitivity and 84.0% specificity. In the validation cohort, the model showed a 75.0% positive predictive value, 86.7% negative predictive value, and 82.6% overall accuracy.

**Conclusion:**

A composite biomarker model based on HDM component responses enabled early prediction of SCIT efficacy, supporting more personalized treatment strategies for AR management.

**Abbreviations**
AIT: Allergen immunotherapy
AR: Allergic rhinitis
AUC: Area under the curve
CRD: Component‐resolved diagnostics
CSMS: Combined Symptom and Medication Score
Der f: *Dermatophagoides farinae*
Der p: *Dermatophagoides pteronyssinus*
HDM: House dust mite
IQR: Interquartile range
ROC: Receiver operating characteristic
SCIT: Subcutaneous immunotherapy
sIgE: Specific immunoglobulin E
sIgG4: Specific immunoglobulin G4
tIgE: Total IgE
TNSS: Total nasal symptom score

## 1 Introduction

Allergic rhinitis (AR), a chronic inflammatory disorder predominantly driven by immunoglobulin E‐mediated type I hypersensitivity reactions upon allergen exposure, manifests clinically as nasal congestion, nasal pruritus, rhinorrhea, and sneezing [1]. AR affects up to 400 million people worldwide [2] and ranks among the most prevalent chronic conditions worldwide, conferring significant socioeconomic burdens through direct healthcare costs and indirect productivity losses [3].

House dust mites (HDM), predominantly *Dermatophagoides farinae* (Der f) and *Dermatophagoides pteronyssinus* (Der p), represents the most prevalent indoor allergens that drive perennial AR. Recent advances in molecular allergology have led to component‐resolved diagnostics (CRD), permitting detailed molecular‐level profiling of individual allergen components [4]. To date, 44 Der f and 40 Der p components have been characterized (data from www.allergen.org). Clinically, Group 1 and 2 allergens (Der f 1, Der f 2, Der p 1, and Der p 2) constitute major sensitization drivers [5]. These components elicit specific immunoglobulin E (sIgE) responses in approximately 80% of sensitized individuals, establishing them as primary diagnostic and therapeutic targets in HDM‐associated AR [6]. Der p 23 is another common component of allergens that is often associated with asthma [7]. Clinically, Der p 5, Der p 7, and Der p 21 are moderately relevant allergens [8]. Der 10, a minor allergen, may trigger cross‐reactive allergic reactions due to its structural similarity to tropomyosin in crustaceans [9]. Therefore, in HDM‐associated AR research, attention has been focused on Der f 1, Der f 2, Der p 1, Der p 2, and Der p 23.

Allergen immunotherapy (AIT) is a unique disease‐modifying intervention for AR [10] that can alter the natural course of IgE‐mediated hypersensitivity [11]. Although AIT has demonstrated clear clinical benefits, individual responses to treatment can vary widely. Studies have shown that a considerable percentage of patients, approximately 20%–50%, exhibit suboptimal outcomes with subcutaneous immunotherapy (SCIT) [12, 13]. The unpredictability of treatment effectiveness, along with factors such as prolonged therapy duration and the risk of adverse effects, has significantly undermined patient confidence and led to poor adherence. Consequently, there is an urgent need to identify reliable predictive biomarkers that can distinguish responders early in SCIT and help refine therapeutic strategies.

Previous studies have shown that patients with higher levels of Der p 1 sIgE at baseline are more likely to respond to SCIT [14]. Moreover, a significant correlation between the serum sIgE/total IgE and the clinical efficacy of AIT has been reported [15]. Moreover, a hallmark of successful AIT is the induction of allergen‐specific IgG4 (sIgG4), which competes with IgE for allergen binding, thereby preventing the formation of allergen‐IgE complexes [16, 17]. Additionally, the ratio of Der p 1 sIgE to sIgG4 has been proposed as a potential biomarker for predicting clinical response to AIT [18].

To date, studies exploring the early prediction of AIT efficacy on the basis of component allergens are limited. In this study, we first aimed to characterize the longitudinal changes in sIgE and sIgG4 to HDM components during AIT. Second, we sought to develop a predictive model based on HDM components to assess the AIT response in a training cohort. Finally, we tested the predictive accuracy of this model in a validation cohort.

## 2 Methods

### 2.1 Study Population

A total of 66 AR patients who received SCIT with a conventional schedule (Alutard SQ, ALK Company, Denmark) at the Eye and ENT Hospital of Fudan University between March 2023 and December 2024 were enrolled in this study. The first 43 AR patients were assigned to the training cohort for model development, whereas the subsequent 23 patients (prospectively enrolled) constituted the validation cohort for evaluation of the predictive performance of the model. AR was diagnosed according to the Allergic Rhinitis and its Impact on Asthma guidelines [10]. Both cohorts followed the same inclusion and exclusion criteria [19]. The study prospectively collected questionnaire data for efficacy evaluation and blood samples at three time points: baseline (V1), 15 weeks (V2, end of the updosing phase), and 1 year (V3). Serum was isolated from whole blood and then cryopreserved at −80°C for subsequent CRD targeting HDM. All patients completed three full rounds of clinical questionnaires and serum sample collection. The study protocol was reviewed and approved by the Ethics Committee of the Eye and ENT Hospital of Fudan University (2023018). Written informed consent was obtained from all participants or their legal guardians (for those under 18 years of age) prior to enrollment in the study.

### 2.2 Immunoglobulin Measurements

Serum total IgE (tIgE), Der p sIgE, and Der f sIgE levels were quantified using ImmunoCAP (Phadia, Sweden), with measurement ranges of 2–5000 kU/L for tIgE and 0.1–100 kUA/L for sIgE; values above the upper limit were recorded as the maximum reportable value. Levels of allergen‐component sIgE and sIgG4 (Der p 1, Der f 1, Der p 2, Der f 2, Der p 5, Der p 7, Der p 10, Der p 21, and Der p 23) were measured using a protein microarray (Zheda Dixun, China), which showed good agreement with the ImmunoCAP assay [20]. Sensitization to HDM component allergens was defined as an sIgE level ≥ 0.35 IU/mL (range: 0.02–100 IU/mL); the sIgG4 level for HDM components was measured within 10–1000 U_A_/mL, with values above the range assigned to the maximum reportable.

### 2.3 Clinical Efficacy Evaluation

The Total Nasal Symptom Score (TNSS) ranges from 0 to 12. The TNSS includes 4‐point scales for four main nasal symptoms, namely, nasal congestion, rhinorrhea, nasal pruritus, and sneezing. The medication score (MS) was graded on a scale of 0–3 points: 0, no medication use; 1, oral and/or topical antihistamines; 2, intranasal corticosteroids with or without antihistamines; and 3, oral corticosteroids with or without intranasal corticosteroids, with or without antihistamines. The Combined Symptom and Medication Score (CSMS) was calculated as previously described and scaled from 0–6 [21]. Responsiveness to SCIT was defined by a reduction in the CSMS relative to baseline values. Patients with a < 50% reduction in the CSMS were classified as nonresponders, whereas patients with a ≥ 50% reduction in the CSMS were classified as responders.

### 2.4 Statistical Analysis

Statistical analyses were conducted using GraphPad Prism 9.0, SPSS 20.0, and R 4.0.0. Categorical variables were compared via the chi‐square test or Fisher's exact test, whereas continuous variables are presented as medians with interquartile ranges (IQRs) and were analyzed using the Mann‒Whitney *U* test. Two‐way repeated‐measures analysis of variance (ANOVA) was used to assess group × time interactions, with Mauchly's test for sphericity and Greenhouse‒Geisser corrections used as needed; Bonferroni‐adjusted post hoc test was applied for within‐group comparisons. Variables with *p* < 0.05 in the univariate analysis were included in the logistic regression models. Model performance was evaluated using receiver operating characteristic (ROC) curve analysis, and the area under the curve (AUC) was calculated. The optimal cutoff point was determined on the basis of Youden's index. Correlations were assessed using Spearman's rank coefficient and visualized in heat maps (Origin 10.1.0). UpSet plots were generated with the UpSetR package (v1.4.0). A *p* value < 0.05 was considered statistically significant.

## 3 Results

### 3.1 Demographic Characteristics of the Participants

Forty‐three AR patients were included in the model development cohort. On the basis of predefined response criteria, after 1 year of SCIT, 18 patients were classified as responders, whereas the remaining 25 patients were categorized as nonresponders. The responders demonstrated significantly higher baseline CSMS than the nonresponders did [3 (IQR: 2.7–3.4) versus 2.7 (IQR: 1.8–2.9), *p* = 0.001]. A total of 23 patients with AR were included in the validation cohort, of whom 8 were classified as responders and 15 were classified as nonresponders. The patients' baseline clinical characteristics were comparable to those of patients in the training cohort. Detailed comparative data are systematically presented in Table 1.

**TABLE 1:** Demographic data and clinical characteristics of participants.

Variables	Training cohort				Validation cohort			
	Responders (*n* = 18)	Non responders (*n* = 25)	Total (*n* = 43)	*p* value	Responders (*n* = 8)	Non responders (*n* = 15)	Total (*n* = 23)	*p* value
Age (year)				0.497				0.470
Median (Q1; Q3)	13.5 (11.0; 16.3)	13.0 (10.0; 16.5)	13.0 (11.0; 16.0)		10.0 (7.0; 23.0)	8.0 (7.0; 12.0)	12.0 (7.0; 26.0)	
Gender				0.181				0.074
Female *n* (%)	5 (27.7)	12 (48.0)	18 (41.9)		1 (12.5)	9 (60.0)	10 (43.5)	
Male *n* (%)	13 (72.2)	13 (52.0)	25 (58.1)		7 (87.5)	6 (40.0)	13 (56.5)	
Body Mass index				0.267				0.629
Mean ± SD	21.0 ± 3.8	19.8 ± 3.1	20.3 ± 3.4		18.8 (16.4; 20.6)	20.1 (16.0; 21.8)	19.7 (16.0; 21.4)	
Disease duration (year)				0.773				0.812
Median(Q1; Q3)	6.5 (4.0; 8.0)	6.0 (4.5; 10.0)	6.0 (4.0; 8.0)		4.0 (3.3; 9.5)	5.0 (3.0; 10.0)	5.0 (3.0; 10.0)	
Monosensitized^a^				0.904				0.369
*n* (%)	15 (83.3%)	20 (80.0%)	35 (81.3%)		7 (87.5%)	10 (66.7%)	17 (73.9%)	
Patients with asthma				0.502				0.781
*n* (%)	0 (0.0%)	2 (8.0%)	2 (4.7%)		2 (25.0%)	3 (20.0%)	5 (21.7%)	
CSMS at baseline				**0.001**				**0.006**
Median (Q1; Q3)	3.0 (2.7; 3.4)	2.7 (1.8; 2.9)	2.7 (2.2; 3.2)		3.5 (3.0; 4.3)	2.7 (2.0; 3.3)	2.8 (2.3; 3.5)	
tIgE at baseline (KU/L)				0.457				0.851
Median (Q1; Q3)	260.0 (155.5; 588.0)	353.2 (135.7; 686.5)	288.4 (158.4; 594.6)		174.0 (74.2; 396.0)	234.0 (88.3; 452.3)	211.0 (88.3; 396.0)	
Der p sIgE at baseline (KUA/L)				0.976				0.776
Median (Q1; Q3)	33.1 (11.76; 53.0)	24.8 (13.5; 66.9)	31.3 (12.6; 60.1)		26.5 (9.3; 57.4)	23.8 (5.8; 56.6)	24.7 (8.2; 56.6)	
Der f sIgE at baseline (KUA/L)				0.917				0.856
Median (Q1; Q3)	42.8 (11.8; 64.4)	38.7 (14.4; 80.5)	41.2 (12.9; 77.1)		25.1 (8.6; 33.3)	30.3 (7.2; 51.6)	25.1 (8.5; 46.4)	

*Note:* Bold indicates *p* value < 0.05 was considered statistically significant between responders and nonresponders.

Abbreviations: CSMS, combined symptom and medication score; Der f, *Dermatophagoides farinae*; Der p, *Dermatophagoides pteronyssinus*.

^a^ No sensitization other than Der p or Der f among the tested allergens.

### 3.2 Clinical Efficacy of SCIT in the Training Cohort

Following 1 year of SCIT, the responders exhibited significant reductions in both the TNSS [6 (IQR: 4–8) versus 4 (IQR: 2–5), *p* = 0.001; Supporting Information S1: Figure S1B] and MS [2 (IQR: 2–2) versus 0 (IQR: 0–0), *p* < 0.001; Supporting Information S1: Figure S1C], with notable improvements in scores across nasal symptom domains, including nasal congestion, rhinorrhea, nasal pruritus, and sneezing severity scores (Supporting Information S1: Figures S1D–G). Notably, after 1 year of SCIT, the responders demonstrated significantly lower MS than the nonresponders did [0 (IQR: 0–0) versus 1 (IQR: 1–1), *p* < 0.001; Supporting Information S1: Figure S1C], indicating that sustained SCIT intervention confers substantial medication‐sparing benefits in patients with a good response.

### 3.3 Baseline Component‐Resolved Sensitization Profiles to HDM Allergens in the Training Cohort

**FIGURE 1:**
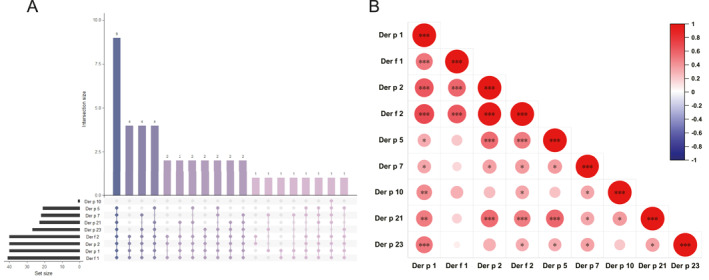
Baseline HDM components sensitization profiles in the training cohort (*n* = 43). (A) UpSet plot shows HDM components cosensitization patterns. (B) Intercomponent correlations was showed via a heatmap. Dot size and color intensity reflect the strength of Spearman's correlation. *p < 0.05, **p < 0.01, ***p < 0.001.

Baseline analysis of HDM component sensitization in the training cohort revealed the following prevalences: Der f 1 (95.3%) > Der p 1 (93.0%) = Der p 2 (93.0%) = Der f 2 (93.0%) > Der p 23 (62.8%) > Der p 21 (53.5%) > Der p 7 (51.2%) > Der p 5 (48.8%) > Der p 10 (2.3%) (Figure 1A). The prevalence of sensitization was similar between the responders and the nonresponders. UpSet plot visualization of HDM component cosensitization patterns demonstrated universal multicomponent sensitization (≥ 2 components). The most prevalent combination, observed in 20.9% (9/43) of patients, was cosensitization to eight components excluding Der p 10 (Figure 1A). Correlation analysis among nine HDM components revealed three distinct coreactive clusters: (1) positive correlations (*ρ* = 0.52–0.97, *p* < 0.001) between Group 1/2 HDM components (Der p 1‐Der f 1‐Der p 2‐Der f 2); (2) mid‐range correlations (*ρ* = 0.49–0.57, *p* < 0.001) linking Der p 2‐Der f 2‐Der p 5‐Der p 21; and (3) a special link (*ρ* = 0.51, *p* < 0.001) between Der p 1 and Der p 23 (Figure 1B).

### 3.4 Changes in the Serum Levels of sIgE to HDM Components During SCIT in the Training Cohort

**FIGURE 2:**
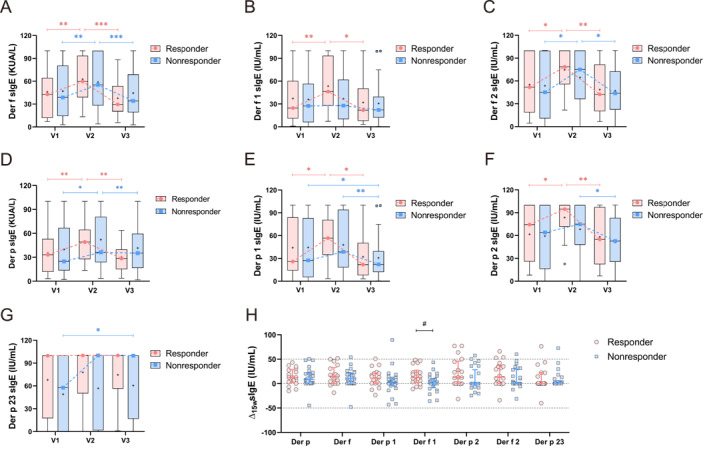
Dynamic changes in serum sIgE to HDM components during SCIT in the training cohort (*n* = 43). (A–G) Longitudinal changes in serum levels of sIgE to HDM components at V1 (baseline), V2 (15‐week follow‐up), and V3 (1‐year follow‐up). Outliers are represented by pink circles or blue squares, with “+” symbols designating mean values. (H) Intergroup comparison of 15‐week changes (Δ [V2–V1]) in serum sIgE levels. The detection range for sIgE to HDM components is 0.02–100 IU/mL. *p < 0.05, **p < 0.01, ***p < 0.001 indicate within‐group comparisons; #*p* < 0.05, ##*p* < 0.01, ###*p* < 0.001 indicate between‐group comparisons.

In both the responders and the nonresponders, serum levels of sIgE to Der f, Der f 2, and Der p exhibited transient elevation at 15 weeks, followed by a sustained reduction at 1 year (Figure 2A,C,D). Only in the responders did serum levels of sIgE to Der f 1, Der p 1, and Der p 2 show a characteristic increasing‒decreasing pattern during SCIT (Figure 2B,E,F). The nonresponders showed a significant decrease in Der p 1 sIgE and an increase in Der p 23 sIgE after 1 year (Figure 2E,G). However, no significant changes in serum levels of sIgE to Der p 5, Der p 7, Der p 10, or Der p 21 were observed throughout the treatment period (Supporting Information S1: Figure S2A‐D). Furthermore, significant differences in Der f 1 sIgE alterations after 15 weeks of SCIT were observed between the responders and the nonresponders (Figure 2H), whereas no intergroup differences in serum levels of sIgE to other HDM components were observed (Figure 2H and Supporting Information S1: Figure S2E). In addition, significant differences in the Der f 1 sIgE/tIgE were observed between groups at both V2 and V3 (Supporting Information S1: Figure S3). The Der p 23 sIgE/tIgE and Der p 23 sIgE/Der p sIgE ratios demonstrated persistent intergroup disparities across all timepoints (Supporting Information S1: Figures S3 and S4).

### 3.5 Changes in Serum Levels of sIgG4 to HDM Components During SCIT in the Training Cohort

**FIGURE 3:**
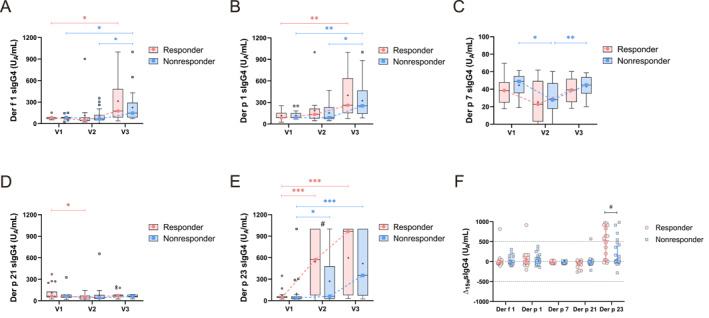
Dynamic changes in serum sIgG4 to HDM components during SCIT in the training cohort (*n* = 43). (A–E) Longitudinal changes in serum levels of sIgG4 to HDM components at V1 (baseline), V2 (15‐week follow‐up), and V3 (1‐year follow‐up). Outliers are represented by pink circles or blue squares, with “+” symbols designating mean values. (F) Intergroup comparison of 15‐week changes (Δ [V2–V1]) in serum sIgG4 levels. The detection range for sIgG4 is 10–1000 U_A_/mL. *p < 0.05, **p < 0.01, ***p < 0.001 indicate within‐group comparisons; #*p* < 0.05, ##*p* < 0.01, ###*p* < 0.001 indicate between‐group comparisons.

We investigated the dynamic changes in serum levels of sIgG4 to HDM components in relation to different SCIT responses. Following 1 year of treatment, significant increases in serum levels of sIgG4 targeting the major allergens Der f 1, Der p 1, and Der p 23 were observed in both the responders and the nonresponders (Figure 3A,B,E). An initial decline followed by an elevation in serum levels of Der p 7 sIgG4 occurred only in nonresponders (Figure 3C). Conversely, a transient reduction in serum levels of Der p 21 sIgG4 at V2 was observed only in the responders (Figure 3D). No changes in sIgG4 to Der f 2, Der p 2, Der p 5 or Der p 10 were seen during treatment (Supporting Information S1: Figures S5A–D). Additionally, serum Der p 23 sIgG4 levels differed significantly between the groups at V2 (Figure 3E). Moreover, following 15 weeks of SCIT, the magnitude of alterations in serum Der p 23 sIgG4 demonstrated significant intergroup disparity between the groups (Figure 3F), whereas changes in sIgG4 to other HDM components were comparable between groups (Figure 3F and Supporting Information S1: Figure S5E).

### 3.6 Changes in HDM Component Serum sIgE/sIgG4 During SCIT in the Training Cohort

**FIGURE 4:**
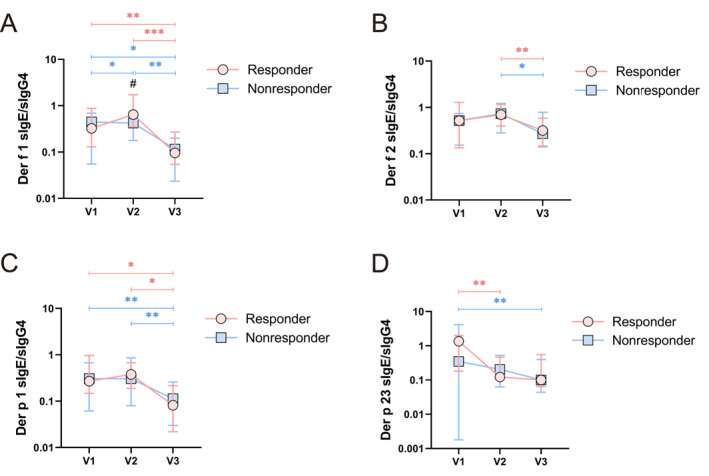
Dynamic changes in serum sIgE/sIgG4 to HDM components during SCIT in the training cohort (*n* = 43). (A–D) Temporal changes in sIgE/sIgG4 to Der f 1, Der f 2, Der p 1, and Der p 23 at V1 (baseline), V2 (15‐week follow‐up), and V3 (1‐year follow‐up) are shown. *p < 0.05, **p < 0.01, ***p < 0.001 indicate within‐group comparisons; #*p* < 0.05, ##*p* < 0.01, ###*p* < 0.001 indicate between‐group comparisons.

We further analyzed the dynamics of HDM component serum sIgE/sIgG4 during SCIT. After 1 year of SCIT, significant reductions in Der f 1 and Der p 1 serum sIgE/sIgG4 were observed in both groups (Figure 4A,C). Both groups exhibited a decrease in Der f 2 sIgE/sIgG4 from V2 to V3 (Figure 4B). Notably, Der p 23 sIgE/sIgG4 decreased significantly as early as V2 in the responders, whereas this reduction was observed only at V3 in the nonresponders (Figure 4D). No significant changes in other HDM component sIgE/sIgG4 were observed (Supporting Information S1: Figures S6A–D). Additionally, a statistically significant difference in Der f 1 sIgE/sIgG4 between the two groups at V2 was observed (Figure 4A).

### 3.7 Development of a Predictive Model Involving HDM Components for 1‐Year SCIT Efficacy

**FIGURE 5:**
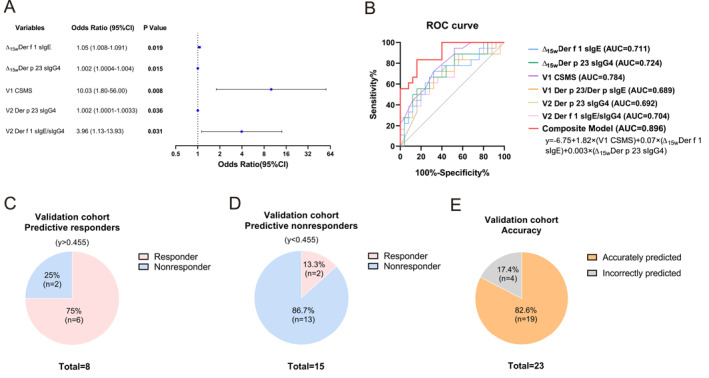
Establishment and validation of an SCIT efficacy prediction model. (A) Univariable logistic regression analysis identifying factors significantly associated with the clinical efficacy of SCIT. (B) ROC curves of individual markers and the composite model. In the validation cohort, (C) actual responders among the predicted responders (*y* > 0.455), (D) actual nonresponders among the predicted nonresponders (*y* < 0.455), and (E) the overall accuracy of the predictions of the composite model.

First, we performed univariable binary logistic regression analysis on variables showing significant differences in the intergroup analyses. The analysis revealed that V1 CSMS, Δ_15w_ Der f 1 sIgE, Δ_15w_ Der p 23 sIgG4, V1 Der p 23 sIgE/Der p sIgE, V2 Der p 23 sIgG4, and V2 Der f 1 sIgE/sIgG4 demonstrated potential associations with SCIT clinical outcomes (Figure 5A). Next, to evaluate the ability of these variables to predict SCIT clinical outcomes, we conducted ROC curve analyses for individual variables (Figure 5B, detailed in Supporting Information S2: Table S1). Finally, the six variables were incorporated into a multivariable binary logistic regression model. The final model identified V1 CSMS, Δ_15w_ Der f 1 sIgE, and Δ_15w_ Der p 23 sIgG4 as independent and significant predictors of SCIT efficacy. The multivariable logistic regression analysis yielded the following composite model: *y* = −6.749 + 1.823 × (V1 CSMS) + 0.068 × (Δ_15w_ Der f 1 sIgE) + 0.003 × (Δ_15w_ Der p 23 sIgG4) (Figure 5B, detailed in Supporting Information S2: Table S2). This model demonstrated superior discriminative capacity, with an AUC of 0.896 (95% CI: 0.804–0.987, detailed in Supporting Information S2: Table S1), achieving 83.3% sensitivity and 84.0% specificity at the optimal cutoff value of 0.455, surpassing the predictive performance of individual variables. We also performed a separate analysis for patients without comorbid asthma, and the results showed similar model performance (Supporting Information S1: Figure S7).

### 3.8 Validation of the Composite Model

To validate the clinical applicability of the composite model, we prospectively enrolled 23 AR patients undergoing SCIT as an independent validation cohort. At the prespecified decision threshold of 0.455, the composite model demonstrated robust predictive performance in the validation cohort. Among patients predicted as responders (*y* > 0.455), 75.0% (6/8) achieved actual clinical improvement, whereas 86.7% (13/15) predicted nonresponders (*y* < 0.455) achieved suboptimal therapeutic outcomes (Figure 5C–D). The overall classification accuracy reached 82.6% (19/23) (Figure 5E).

## 4 Discussion

HDM was recognized as the most prevalent etiological source of allergic diseases [22]. CRD plays a pivotal role in identifying major allergenic proteins and guiding AIT [23, 24]. Sensitized individuals exhibit diverse component‐specific IgE profiles with implications for treatment. In this study, the predominant sensitizing components in HDM‐sensitized AR patients were identified as Der p 1, Der f 1, Der p 2, Der f 2, and Der p 23, which exhibited significant intercorrelations. Furthermore, longitudinal monitoring of levels of sIgE and sIgG4 to nine HDM components revealed that Δ_15w_ Der f 1 sIgE, Δ_15w_ Der p 23 sIgG4, V1 Der p 23 sIgE/Der p sIgE, V2 Der p 23 sIgG4, and V2 Der f 1 sIgE/sIgG4 were associated with favorable treatment outcomes. Crucially, a predictive model integrating Δ_15w_ Der f 1 sIgE, Δ_15w_ Der p 23 sIgG4 and V1 CSMS demonstrated robust efficacy in predicting the SCIT therapeutic response at the 1‐year endpoint, with an accuracy of 82.6% in the validation cohort.

Group 1 and 2 HDM components are widely recognized as clinically predominant allergens [25, 26, 27]. Our findings are consistent with those of previous reports, with over 93% of participants sensitized to at least one of the four components [28, 29]. Despite the high sequence homology (81%) of Der p 1 and Der f 1 [9], Der f 1 is highly immunodominant in South Korea, Turkey, and Eastern China [28, 30, 31, 32, 33]. The strong correlation between Der p 2 and Der f 2 aligns with our results (*ρ* = 0.97, *p* < 0.05) [32, 34]. Emerging evidence links Der p 23 to AR [29], and Der p 23 is associated with severe type [35]. In our cohort, all participants were sensitized to more than two HDM components, yielding 19 distinct profiles. However, no specific pattern or single‐component sensitization predicted efficacy, which aligns with prior studies [14, 27]. These results highlight the limitation of relying solely on individual component sensitization status to predict SCIT responsiveness.

We mapped longitudinal profiles of sIgE to HDM components at baseline, at the end of the updosing phase, and at 1 year during SCIT. Consistent with previous findings [36, 37, 38], levels of sIgE to Der p and Der f exhibited a characteristic biphasic pattern of initial elevation followed by a gradual decline. This trend was similarly observed for major allergenic components (Der p 1, Der p 2, Der f 1, and Der f 2) and remained comparable between the responders and the nonresponders. Although a prior study mapping sIgE profiles at baseline, 6 months, and 18 months revealed analogous trends, the observed changes lacked statistical significance [37]. This discrepancy may stem from the 6‐month sampling window capturing the postpeak phase in the prior study, whereas our 15‐week timepoint achieved statistical significance, suggesting that this interval represents a critical juncture in SCIT‐induced immunomodulation. Notably, cluster immunotherapy shows earlier peaks at 5 weeks [18], a phenomenon attributable to the accelerated 7‐week dose escalation schedule implemented for this type of therapy. In contrast, conventional SCIT regimens do not lead to a significant increase in sIgE levels at 7 weeks [36]. Consistent with the findings of multiple studies [14, 27, 37, 39], cross‐sectional sIgE measurements at baseline, 15 weeks, and 1 year failed to distinguish responders. However, we identified a distinctive immunological signature: the responders presented significantly greater Der f 1 sIgE level increases at 15 weeks than the nonresponders did. No significant changes were observed for minor allergens (Der p 5, Der p 7, Der p 10, and Der p 21), which aligns with the established literature [36, 39].

While findings from some studies have suggested that the baseline sIgE/tIgE may be indicative of AIT efficacy, with higher ratios predicting superior outcomes [15], other studies have revealed only modest predictive utility [12, 40, 41]. In our study, the baseline sIgE/tIgE did not significantly differ between the responders and the nonresponders. Although we noted higher Der f 1 and Der p 23 sIgE/tIgE in the responders at certain timepoints, regression analysis revealed no statistically significant associations of these ratios with clinical outcomes.

In AIT, sIgG4 induction is widely regarded as a critical immune response biomarker [17, 42, 43]. However, its predictive value remains debated; while certain study findings support the relevance of sIgG4 induction in predicting treatment outcomes [36, 39, 44], other studies revealed that the prognostic significance of sIgG4 induction may be overestimated [37, 39, 43]. Intriguingly, the level of Der p 23 sIgG4 was elevated early in our study (by 15 weeks), with the responders showing a markedly greater increase. This divergence was evident in both the absolute levels and magnitude of change at the 15‐week time point, suggesting that robust Der p 23 sIgG4 induction during the updosing phase may favor therapeutic success. Our findings suggest that early elevation of Der p 23 sIgG4 could serve as a predictive biomarker for optimized SCIT outcomes. We also observed a transient decrease in Der p 7 and Der p 21 sIgG4 levels during the first 15 weeks, which may reflect immune fluctuations occurring in the dose‐escalation phase as well as the low abundance of these non‐major allergens in dust mite extracts. Notably, their levels returned to baseline by 1 year, highlighting the need for larger studies with extended follow‐up to clarify their long‐term dynamics.

Huang et al. proposed that baseline Der p 1 sIgE/sIgG4 might predict clinical responses to cluster SCIT, demonstrating a moderate correlation with symptom improvement rates [18]. In our cohort, the baseline Der p 1 sIgE/tIgE was not significantly different between the groups. However, the V2 Der f 1 sIgE/sIgG4 was significantly greater in responders than in nonresponders and was correlated with treatment efficacy according to the univariable analysis, although the ROC curve analysis revealed only moderate predictive power of this variable (AUC = 0.704).

ROC curve analysis revealed that the composite model comprising the V1 CSMS, Δ_15w_ Der f 1 sIgE, and Δ_15w_ Der p 23 sIgG4 was the optimal predictor of a good 1‐year response to SCIT (AUC = 0.896). Moreover, our results suggest that a higher baseline CSMS is predictive of better therapeutic outcomes, which is consistent with previous reports indicating that elevated baseline symptom medication scores may be correlated with improved treatment efficacy [45]. Interestingly, we found that greater increases in the levels of Der f 1 sIgE and Der p 23 sIgG4 during the updosing phase were associated with a greater likelihood of achieving favorable treatment outcomes. This model demonstrated robust discriminative performance in our prospectively collected validation cohort, suggesting its practical utility in clinical practice.

Although we established a relatively robust predictive model, our study also has certain limitations. First, given the small number of responders, there is a potential risk of model overfitting. Future studies with larger cohorts are necessary to enhance the reliability of the results. Second, as this was a single‐center study, external validation was lacking. Therefore, multicenter studies are warranted to confirm the robustness and generalizability of our findings. Third, the predictive performance of this model is limited to a one‐year follow‐up, and further studies with longer‐term follow‐up are needed to assess its value in predicting sustained remission and long‐term safety.

In conclusion, we observed that the levels of sIgE and sIgG4 to major HDM components initially increased but then subsequently decreased over 1 year of SCIT. Furthermore, the combination of 15‐week changes in Der f 1 sIgE, Der p 23 sIgG4 and baseline CSMS effectively predicted 1‐year efficacy and was prospectively validated. Our findings support the use of molecular diagnostics to advance precision medicine in allergology.

## Author Contributions

**Shuying Li:** writing – original draft, visualization, writing – review and editing, software. **Yue Ma:** writing – review and editing, writing – original draft, validation, formal analysis. **Jiaying Li:** data curation, investigation. **Xian Feng:** data curation. **Fang Xiong:** data curation. **Miaomiao Han:** conceptualization, project administration. **Huabin Li:** conceptualization, funding acquisition, resources. **Hongfei Lou:** conceptualization, methodology, funding acquisition, supervision.

## Conflicts of Interest

The authors declare no conflicts of interest.

## Supporting information


Supporting Information S1

Supporting Information S2

## Data Availability

The data that support the findings of this study are available from the corresponding author upon reasonable request.
