# Supplementary material for: Early Changes in House Dust Mite Component Specific Immunoglobulin Levels Predict the One‐Year Efficacy of Allergen Immunotherapy in Patients With Allergic Rhinitis
Source: Clin Transl Allergy. 2025 Sep 8;15(9):e70099. doi: 10.1002/clt2.70099 (PMC12416371; doi:10.1002/clt2.70099)
Supplement: Supplementary file 1 — Supporting Information S1 [file CLT2-15-e70099-s002.docx]

**Supplementary Figure**

**
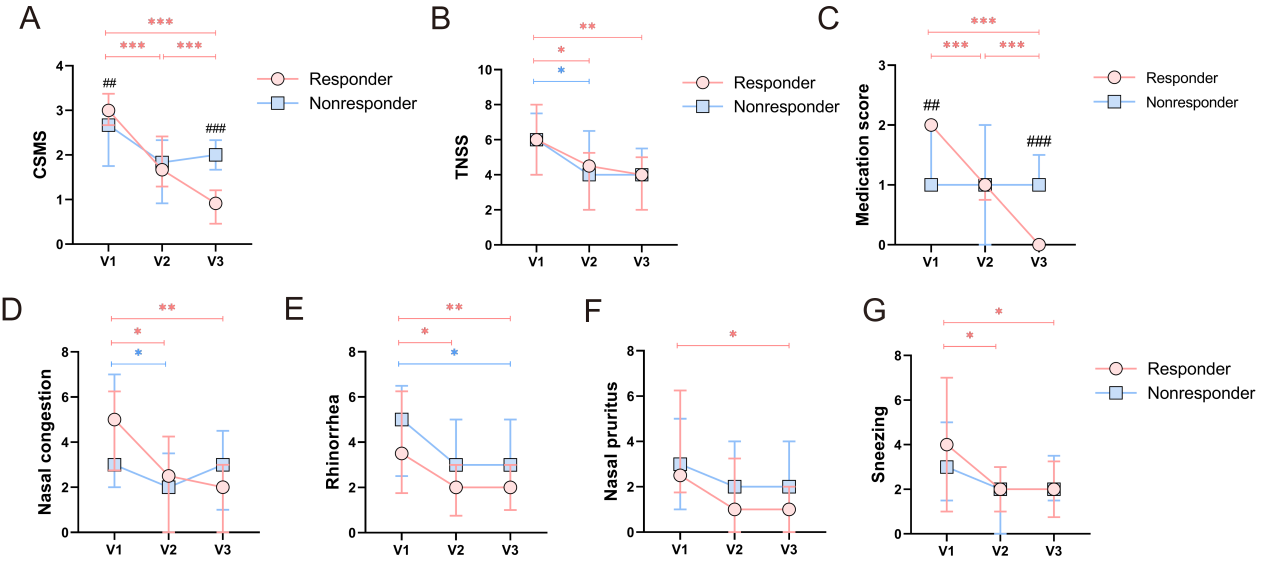
**

**Figure S1. Dynamic changes of symptoms during SCIT in the training cohort (n = 43).** Dynamics of the (A) CSMS, (B) TNSS, (C) MS, and VAS scores for (D) nasal congestion, (E) rhinorrhea, (F) nasal pruritus, and (G) sneezing at baseline (V1), 15 weeks (V2), and 1 year (V3) in the responders (n = 18) and the nonresponders (n = 25). *p < 0.05, **p < 0.01, ***p < 0.001 indicate within-group comparisons; #p < 0.05, ##p < 0.01, ###p < 0.001 indicate between-group comparisons. CSMS, Combined Symptom and Medication Score; TNSS, Total Nasal Symptom Score; MS, medication score; VAS, Visual Analog Scale.


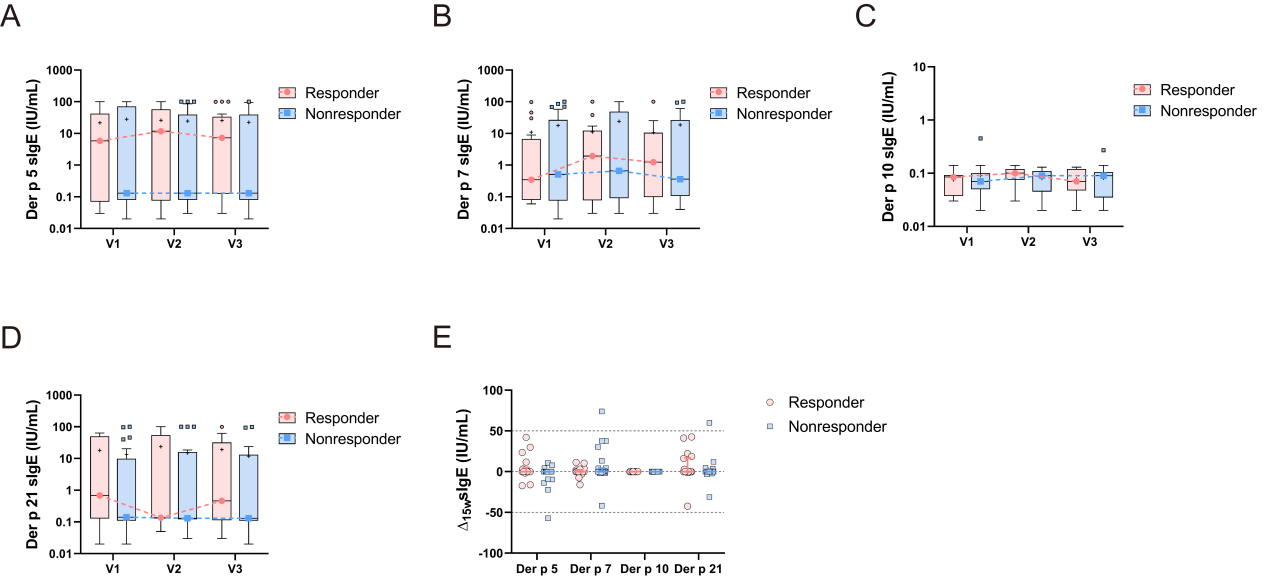


**Figure S2.** **Dynamic changes in serum sIgE to HDM components during SCIT in the training cohort (n = 43).** (A-D) Longitudinal changes in serum sIgE to HDM components at V1 (baseline), V2 (15-week follow-up), and V3 (1-year follow-up) in the training cohort (n = 43). (E) Intergroup comparison of 15-week changes (Δ[V2–V1]) in serum sIgE levels.


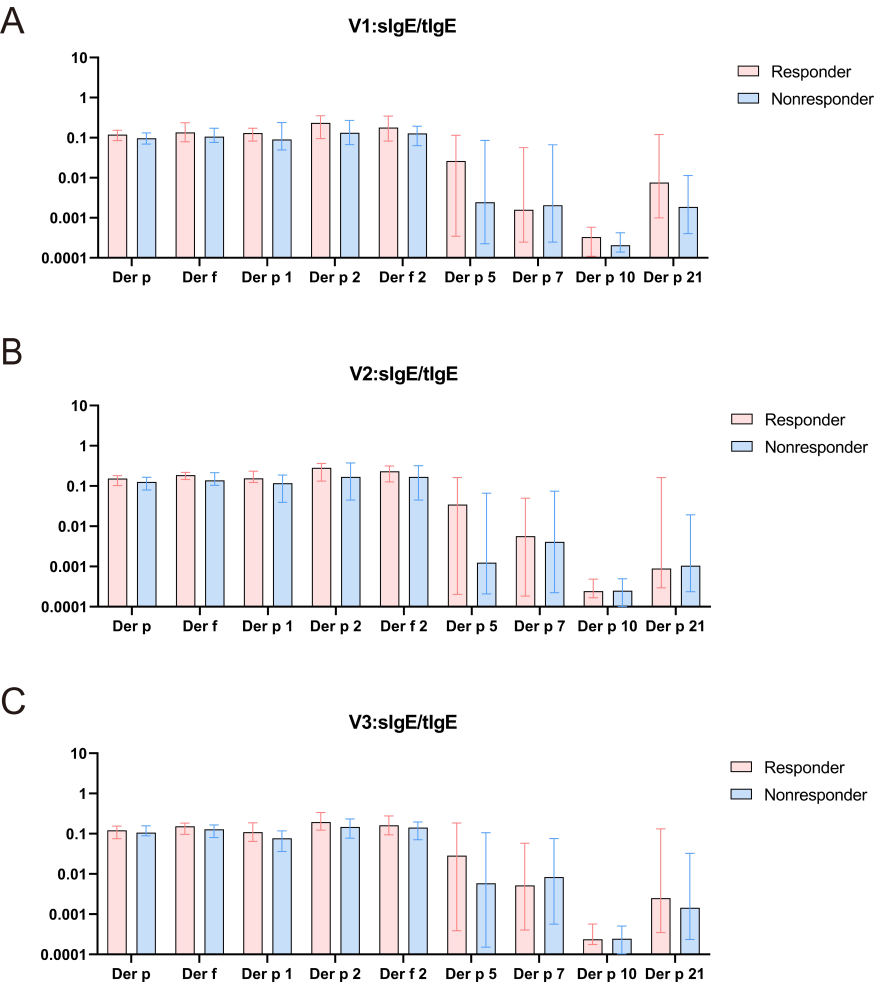


**Figure S3. Comparative analysis of the ratio of HDM component sIgE to tIgE** at (A) baseline, (B) 15-week, and (C) 1-year follow-ups between the responders (n=18) and the nonresponders (n=25). #p < 0.05, ##p < 0.01, ###p < 0.001 indicate between-group comparisons.


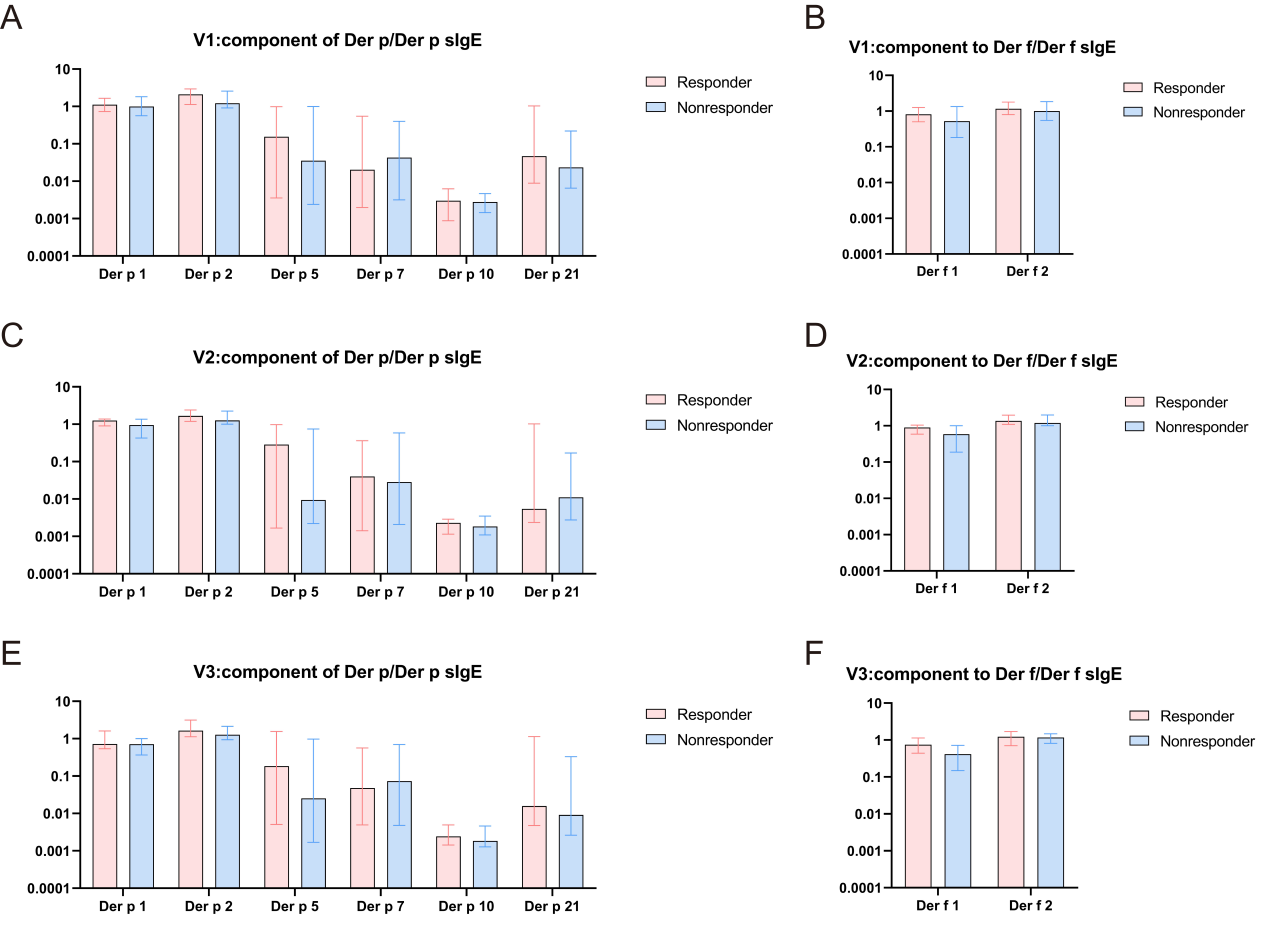


**Figure S4.** **Comparative analysis of the ratio of HDM component sIgE to Der p sIgE or Der f sIgE between the responders (n=18) and the nonresponders (n=25).** Der p component sIgE/Der p sIgE at (A) baseline, (C) 15-week, and (E) 1-year follow-ups. Der f component sIgE/Der f sIgE at (B) baseline, (D) 15-week, and (F) 1-year follow-ups. #p < 0.05, ##p < 0.01, ###p < 0.001 indicate between-group comparisons.


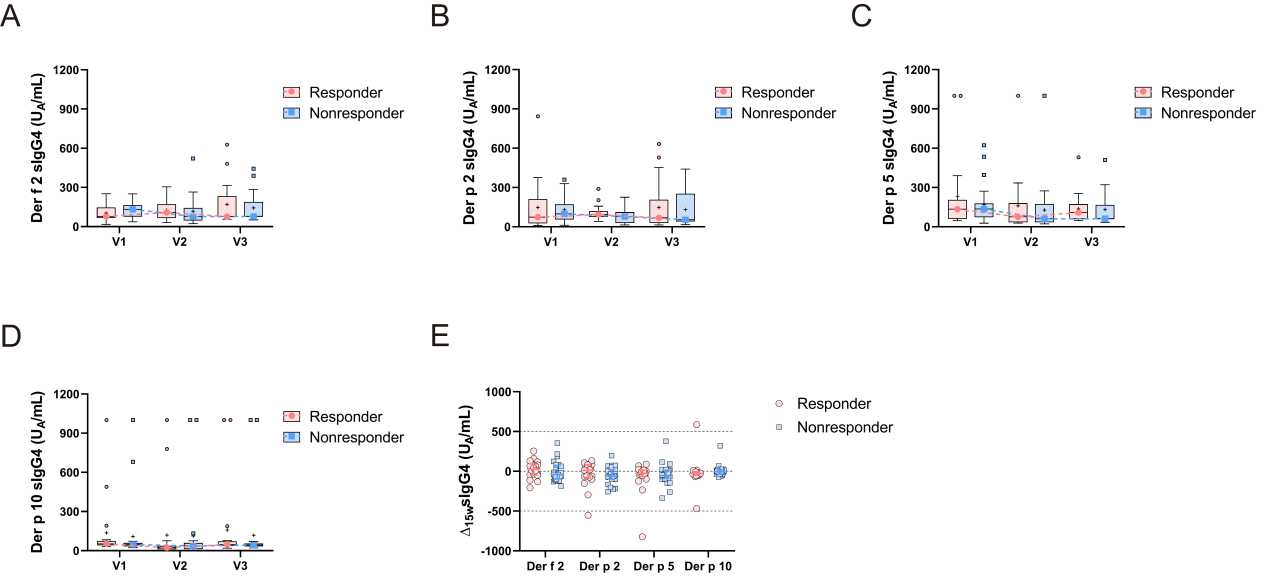


**Figure S5.** **Dynamic changes in serum sIgG4 to HDM components during SCIT in the training cohort (n = 43).** (A-D) Longitudinal changes in serum levels of sIgG4 to HDM components at V1 (baseline), V2 (15-week follow-up), and V3 (1-year follow-up) in the training cohort (n = 43). (E) Intergroup comparison of 15-week changes (Δ[V2–V1]) in serum sIgG4 levels.

**
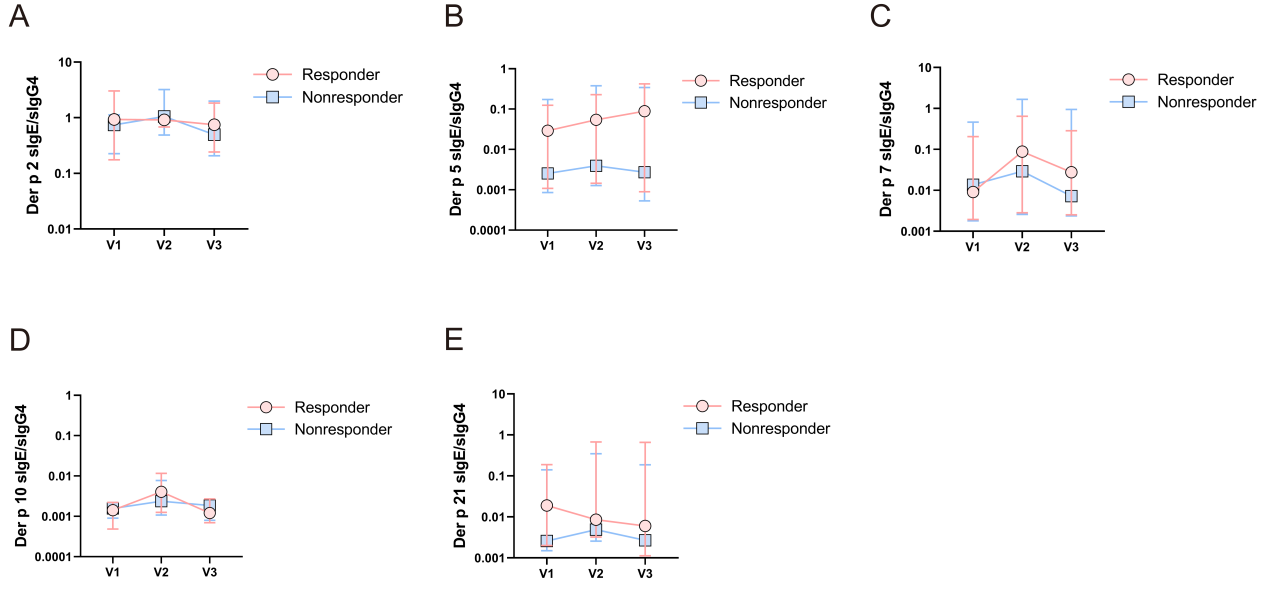
**

**Figure S6.** **Dynamic changes in serum sIgE/sIgG4 to HDM components during SCIT in the training cohort (n = 43).** Temporal changes in sIgE/sIgG4 for (A) Der p 2, (B) Der p 5, (C) Der p 7, (D) Der p 10 and (E) Der p 21 at V1 (baseline), V2 (15-week follow-up), and V3 (1-year follow-up) are shown.


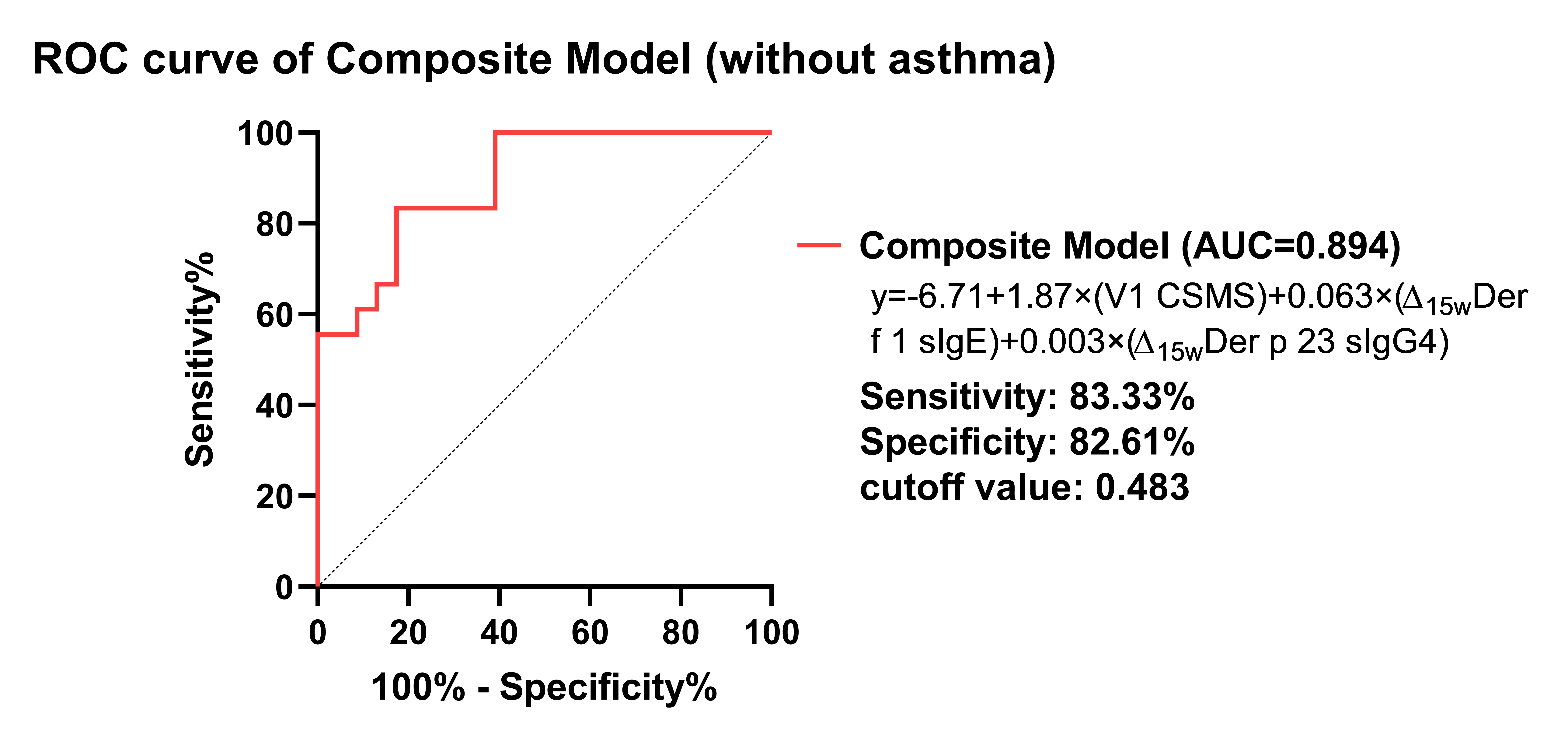


**Figure S7. The ROC curve of the composite model in the non-asthmatic subgroup.**
