# Supplementary material for: Early Changes in House Dust Mite Component Specific Immunoglobulin Levels Predict the One‐Year Efficacy of Allergen Immunotherapy in Patients With Allergic Rhinitis
Source: Clin Transl Allergy. 2025 Sep 8;15(9):e70099. doi: 10.1002/clt2.70099 (PMC12416371; doi:10.1002/clt2.70099)
Supplement: Supplementary file 2 — Supporting Information S2 [file CLT2-15-e70099-s001.docx]

**Supplementary Table**

**Table S1. AUCs for variables potentially predictive 1-year SCIT efficacy.**

| **Predictors** | **AUC** | **S.E.** | **p Value** | **95%CI** |
| --- | --- | --- | --- | --- |
| ∆15wDer f 1 sIgE | 0.711 | 0.081 | 0.019 | 0.552-0.870 |
| ∆15wDer p 23 sIgG4 | 0.724 | 0.080 | 0.013 | 0.568-0.881 |
| V1 CSMS | 0.784 | 0.068 | 0.002 | 0.651-0.918 |
| V1 Der p 23/Der p sIgE | 0.689 | 0.086 | 0.036 | 0.521-0.857 |
| V2 Der p 23 sIgG4 | 0.692 | 0.083 | 0.033 | 0.529-0.856 |
| V2 Der f 1 sIgE/sIgG4 | 0.704 | 0.081 | 0.024 | 0.545-0.864 |
| Composite Model | 0.896 | 0.047 | <0.001 | 0.804-0.987 |

CSMS, combined symptom and medication score; Der p, *Dermatophagoides pteronyssinus*; Der f, *Dermatophagoides farinae*.

**Table S2. Variables information of the composite model calculated using the multivariable logistic regression equation.**

| **Variables** | **β** | **S.E.** | **p Value** | **OR** | **95%CI** |
| --- | --- | --- | --- | --- | --- |
| V1 CSMS | 1.823 | 0.906 | 0.044 | 6.193 | 1.049-36.584 |
| ∆_15w_Der f 1 sIgE | 0.068 | 0.032 | 0.036 | 1.070 | 1.004-1.141 |
| ∆_15w_Der p 23 sIgG4 | 0.003 | 0.001 | 0.034 | 1.003 | 1.000-1.006 |

CSMS, combined symptom and medication score; Der p, *Dermatophagoides pteronyssinus*; Der f, *Dermatophagoides farinae*.
